# Scrambler Therapy for the Treatment of Pain in Schwannomatosis

**DOI:** 10.7759/cureus.23124

**Published:** 2022-03-13

**Authors:** Tyler Murphy, Michael Erdek, Thomas J Smith

**Affiliations:** 1 Medicine, Johns Hopkins School of Medicine, Baltimore, USA; 2 Pain Medicine, Johns Hopkins School of Medicine, Baltimore, USA; 3 Oncology, Hospice and Palliative Medicine, Sidney Kimmel Comprehensive Cancer Center, Johns Hopkins Medicine, Baltimore, USA

**Keywords:** neurofibromatosis, neuropathic pain, schwannomatosis, neuromodulation, scrambler therapy

## Abstract

Schwannomatosis patients (SP) suffer from chronic nerve pain that is often inadequately relieved. Scrambler therapy (ST) can relieve neuropathic pain quickly, safely, and inexpensively. We successfully treated a patient who had disabling leg pain with five daily sessions of ST, each for 40 minutes. She had complete relief of pain and hyperalgesia, with return to normal function, by day 5, that has persisted for at least three weeks. This article briefly describes Schwannomatosis, scrambler therapy, and the need for further research to ascertain the best way to use this neuromodulation.

## Introduction

Schwannomatosis is a condition most frequently characterized by the development of multiple schwannomas throughout the body, and the most common presenting complaint is chronic neuropathic pain [1]. Classical conservative management of chronic neuropathic pain associated with schwannomatosis involves neuropathic agents including gabapentin, amitriptyline, and pregabalin. For schwannomas causing functional limitations, or for patients with refractory pain and/or medication intolerance, surgical resection of the offending lesion remains an effective, albeit more invasive option [2].

Scrambler therapy is a relatively new neuromodulatory treatment approach, which has been shown to provide relief from neuropathic pain [3,4]. Scrambler therapy is believed to be effective by acting on the afferent information aspects of pain, which is accomplished by replacing endogenous pain signals with synthetic signals that travel along the same nervous pathways. These synthetic signals are transmitted via topical electrical stimulation channels which may interact with the surface receptors of C fibers [5]. Through repeated treatments, these synthetic signals can potentially cause a type of “retraining” of the brain, leading to a decrease or resolution of the targeted pain. By nature of its inexpensive minimally invasive methods, scrambler therapy has the potential to be another conservative modality of pain relief for patients with schwannomatosis.

A preprint of this article was previously published in Research Square.

## Case presentation

This 48-year-old woman with known schwannomatosis since age 40 presented with pain in the right anterior thigh and groin. She reported that the pain felt the exact same in quality and location as when she was found to have schwannomas on her left obturator nerve in 2014; then, she underwent left cryoablation of the schwannoma with significant ongoing relief. In the last year, the episodes of right-sided stabbing pain had increased in frequency, and occurred 5-6 times a day with some episodes lasting hours. The pain was worse with lying on her right side, and alleviated with putting more weight on her left side when sitting. She denied any numbness or tingling. She did report some weakness of her right leg, but was able to ambulate without any assistance and has not had any recent falls. She denied any saddle anesthesia or urinary or fecal incontinence.

**Figure 1:**
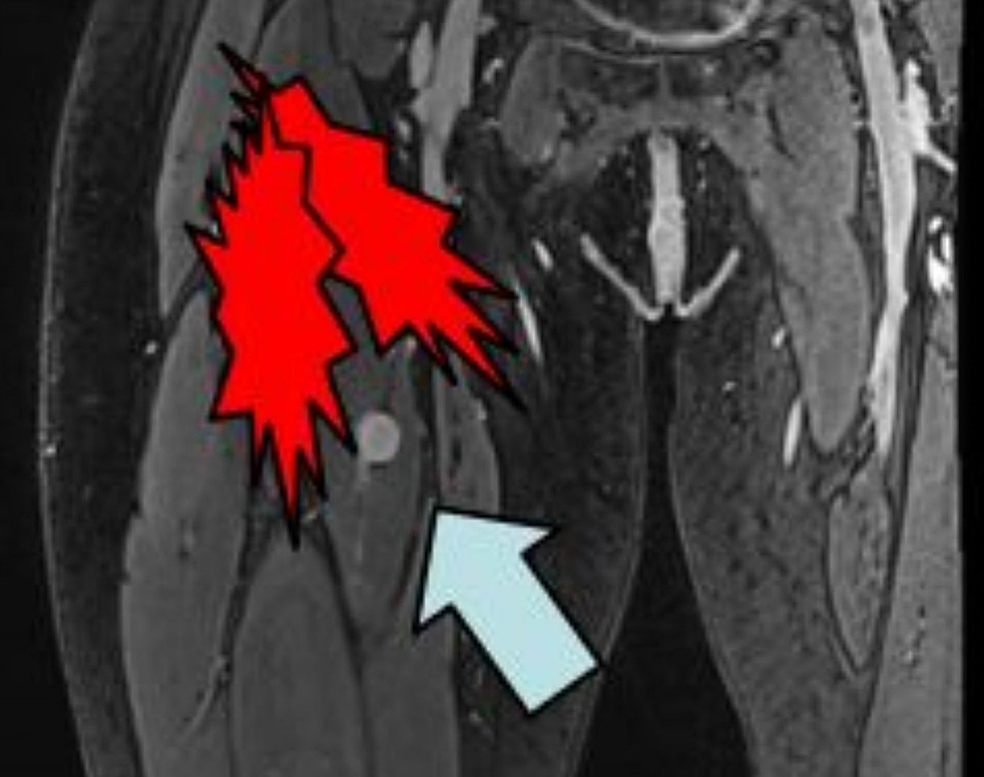
Location of the most visible schwannoma and sites of pain

The tumor in the right quadriceps muscle appeared deep, with a second tumor more medial and proximal tumor and less distinct (Figure 1). Gabapentin at effective doses made her feel overly sedated and “foggy-minded.” She had inadequate benefit from bupropion and nortriptyline prescribed for depression, low-dose naltrexone, celecoxib, or ketorolac. A diagnostic right obturator nerve block with bupivacaine and steroids gave partial relief but also motor paralysis for 24 hours, so she was not considered a good candidate for ablation. Prior to a trial of spinal cord stimulation, she was referred for scrambler therapy.

**Figure 2:**
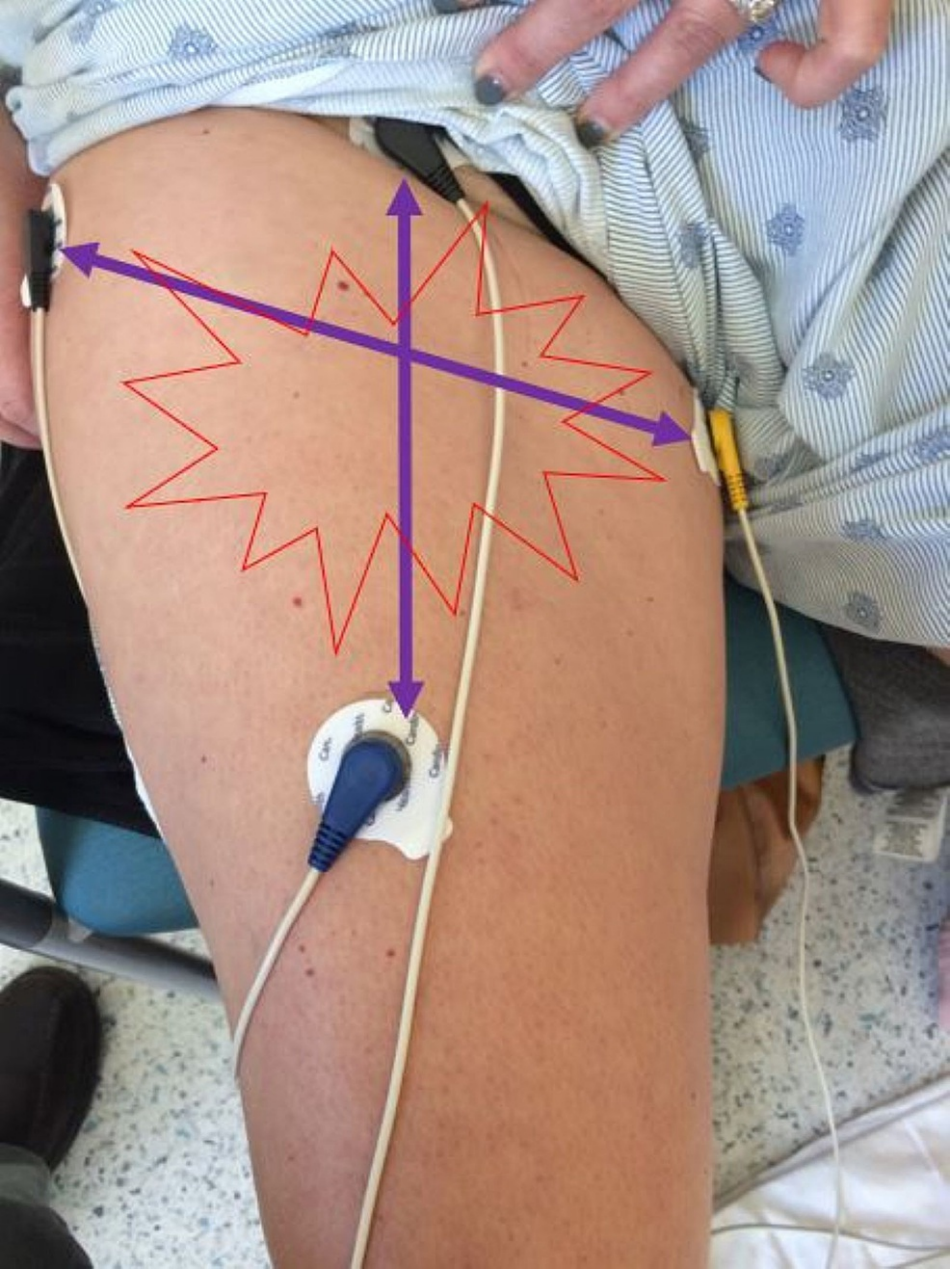
Placement of scrambler therapy electrodes, each about 2 cm beyond the area of pain

**Figure 3:**
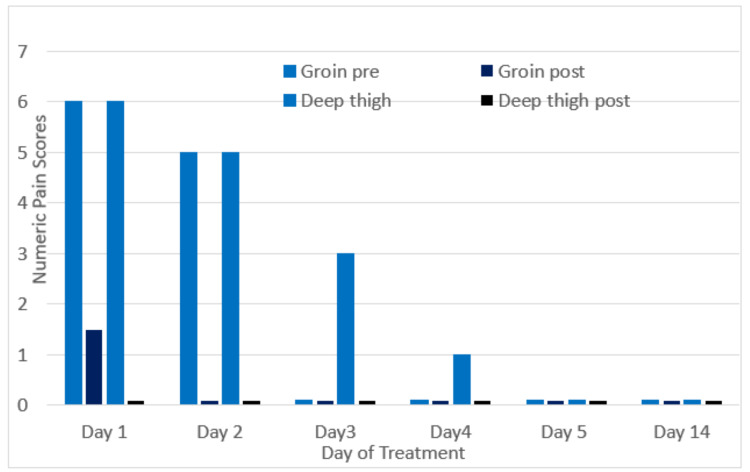
Pain response to scrambler therapy (0 is represented as 0.1 to show on the graph)

Electrodes were placed vertically 2 cm above and below the area of the pain on the quadriceps area, and diagonally across the L1 (medially) and L2 (laterally) dermatomes (Figure 2). She was treated with five 35-minute sessions of scrambler therapy, with the stimulation increased slowly to tolerance and replacement of the pain sensation with the scrambler sensation. Her pain rapidly resolved and has stayed low for over three weeks (Figure 3). She was able to resume normal activities like driving, cooking, and even skiing. If the pain returns, experience shows that “booster” sessions are effective, often taking fewer sessions with the relief lasting longer [6].

Full written permission was obtained to use her story and photographs, and Johns Hopkins does not require IRB approval for three or fewer patient cases.

## Discussion

Scrambler therapy has been used in Europe since 2002 [3], and is effective in other types of neuropathy including transverse myelitis, neuromyelitis optica, chemotherapy-induced neuropathy, Dejerine-Roussy syndrome, low back pain, post-herpetic neuropathy, and others [7-13]. We report here the first case of schwannomatosis pain successfully treated with Scrambler therapy, that gave quick and long-lasting relief and enabled the person to resume full function. Scrambler therapy is becoming more available, is easy to do in the office, and can be reasonably priced. It compares favorably with other treatments such as spinal cord stimulation (over $100,000 per patient, including the trial) and even drugs such as Cymbalta® (Duloxetine) at $122/month generic and $300+/month brand name when used for years (GoodRx®, accessed January 4, 2022). Again, it is non-invasive, no serious side effects have been reported in two decades of use, and desensitization does not occur [3].

The exact mechanism of ST activity is not known, as it was developed without any animal testing, and no one has done nerve mapping experiments. It was invented by Giuseppe Marineo at the University of Rome Tor Vergata after years of research [3]. The most similar treatment, spinal cord stimulation, has multiple potential mechanisms of action but the primary mode is still unproven. Scrambler therapy is thought to capture the c-fiber nerves, and replace the chronic pain stimulus with “non-pain” information, thus reducing or resetting central sensitization and “wind up”. The area being treated does not feel anesthesia, just an analgesic sensation often described as being bitten by electrical ants. In practice, this is done with two questions: “Tell me when you feel something” (at the start) and “Tell me when ‘enough is enough, or when the pain is replaced by the scrambler sensation’”, when adjusting the stimulus upward. It is critically important to stay off the painful areas, in order not to reinforce the pain message.

Scrambler therapy has systemic actions as well as local. In the randomized trial of ST for low back pain, hypersensitivity to cold, heat, and pressure was reduced not only in the treated low back area, but on the arm and the posterior thorax. In addition, the levels of inflammatory neuropeptides found in the blood, such as nerve growth factor (NGF), were reduced significantly [12].

## Conclusions

While schwannomatosis is relatively rare, other forms of neurofibromatosis cause similar problems, and pain is often a lifelong burden. Surgery and invasive blocks run the risk of motor injury and may leave large areas with no feeling. Scrambler therapy does not cause anesthesia in the treated area, and sensation remains normal. Treatment can be repeated as often as necessary. In the words of the inventor, Giuseppe Marineo, and in our own experience, “Once a responder, always a responder.” We have patients who have remained pain free for at least seven years. We believe further research is indicated to determine how best to use this new modality in schwannomatosis and related disorders.

## References

[1] Mansouri S, Suppiah S, Mamatjan Y (2021). Epigenomic, genomic, and transcriptomic landscape of schwannomatosis. Acta Neuropathol.

[2] Farschtschi S, Mautner VF, Lawson McLean AC, Schulz A, Friedrich RE, Rosahl SK (2020). The neurofibromatoses. Dtsch Arztebl Int.

[3] Marineo G (2019). Inside the scrambler therapy, a noninvasive treatment of chronic neuropathic and cancer pain: from the gate control theory to the active principle of information. Integr Cancer Ther.

[4] Kashyap K, Bhatnagar S (2020). Evidence for the efficacy of scrambler therapy for cancer pain: a systematic review. Pain Physician.

[5] Majithia N, Smith TJ, Coyne PJ (2016). Scrambler therapy for the management of chronic pain. Support Care Cancer.

[6] Smith TJ, Auwaerter P, Knowlton A, Saylor D, McArthur J (2017). Treatment of human immunodeficiency virus-related peripheral neuropathy with scrambler therapy: a case report. Int J STD AIDS.

[7] Mealy MA, Newsome SD, Kozachik SL, Levy M, Smith TJ (2019). Case report: Scrambler therapy for treatment-resistant central neuropathic pain in a patient with transverse myelitis. Int J MS Care.

[8] Mealy MA, Kozachik SL, Cook LJ (2020). Scrambler therapy improves pain in neuromyelitis optica: a randomized controlled trial. Neurology.

[9] Loprinzi C, Le-Rademacher JG, Majithia N (2020). Scrambler therapy for chemotherapy neuropathy: a randomized phase II pilot trial. Support Care Cancer.

[10] D'Amato SJ, Mealy MA, Erdek MA, Kozachik S, Smith TJ (2018). Scrambler therapy for the treatment of chronic central pain: a case report. A A Pract.

[11] Christo PJ, Kamson DO, Smith TJ (2020). Treatment of Déjerine-Roussy syndrome pain with scrambler therapy. Pain Manag.

[12] Starkweather AR, Coyne P, Lyon DE, Elswick RK Jr, An K, Sturgill J (2015). Decreased low back pain intensity and differential gene expression following Calmare®: results from a double-blinded randomized sham-controlled study. Res Nurs Health.

[13] Smith TJ, Marineo G (2018). Treatment of postherpetic pain with scrambler therapy, a patient-specific neurocutaneous electrical stimulation device. Am J Hosp Palliat Care.

